# Medullary epithelium-free areas in the rat thymus are specialized niches enriched for mature thymocytes and distinct stromal subsets

**DOI:** 10.3389/fimmu.2026.1945985

**Published:** 2026-10-14

**Authors:** Yasushi Sawanobori, Tadayuki Ogawa

**Affiliations:** 1 Department of Anatomy, Dokkyo Medical University, Mibu, Tochigi, Japan; 2 Laboratory for Molecular Pathobiology, Research Center for Advanced Medical Science, Dokkyo Medical University, Mibu, Tochigi, Japan

**Keywords:** bioinformatics, fibroblast, scRNA-seq, spatial transcriptomics, thymic mesenchymal cells, thymocyte, thymus

## Abstract

The thymic medulla provides the microenvironment for negative selection, late thymocyte maturation, and thymocyte egress, and is generally characterized by widespread distribution of medullary thymic epithelial cells (mTECs). In contrast, the rat thymic medulla contains medullary epithelium-free areas (mEFAs), but the cellular composition and functional significance of these regions remain unclear. Here, to address these questions, we combined spatial transcriptomic data with thymocyte and stromal scRNA-seq reference datasets using robust cell-type decomposition (RCTD) to characterize mEFAs. These analyses revealed that more mature phenotypes within the CD4SP, CD8SP, and regulatory T-cell lineages were preferentially localized in mEFAs, whereas immature SP subsets were enriched in medullary epithelium-containing areas. The newly identified rat thymic mesenchymal cell subsets TMC3 and TMC4 were also enriched in mEFAs. These subsets were broadly similar to mouse medullary fibroblasts but displayed distinct predicted interactions with SP thymocytes, including costimulatory molecule–receptor, chemokine–receptor, and ECM–integrin axes. In addition, venous endothelial cells (vECs) expressing portal endothelial cell markers were enriched in mEFAs. *Spns2*, which encodes a transporter that releases S1P from cells into the extracellular space, was preferentially expressed in both TMC4 and vEC subsets, suggesting that these cells may contribute to the formation of a local S1P gradient within mEFAs. These findings suggest that mEFAs may be specialized medullary niches associated with stromal organization, the localization of mature thymocytes, and thymic egress.

## 1 Introduction

The thymus is the primary organ for T cell development. Following positive selection, thymocytes (T cell progenitors) undergo further selection in the thymic medulla, where self-reactive cells are eliminated through negative selection (1, 2). During this process, thymocytes are exposed to tissue-restricted antigens (TRAs) presented by medullary thymic epithelial cells (mTECs) and dendritic cells (DCs). Thymocytes that bind these self-antigens with excessively high affinity are deleted, thereby helping to prevent autoimmune diseases. Consistent with this central role, mTECs are distributed throughout the thymic medulla in humans and mice.

In contrast, medullary epithelium-free areas (mEFAs) are present in rats and CD-1 mice (3–5). Given the importance of mTECs in negative selection, the presence of such areas without overt pathology is difficult to explain. As a preliminary step toward identifying stromal cell subsets localized in mEFAs, we performed immunohistochemistry (IHC) and found mEFA-localized PDGFRβ expression. Because PDGFRβ is a marker for thymic fibroblasts (6), this finding led us to consider fibroblasts, or mesenchymal subsets including fibroblasts, as candidate stromal cells enriched in mEFAs.

Although mTECs are indispensable for T cell development, other stromal populations, including mesenchymal cells and vascular endothelial cells (ECs), also make major contributions to thymic structure and function (7, 8). Mesenchymal lineage cells, including fibroblasts, adventitial cells, and pericytes, are essential during thymic organogenesis. Neural crest-derived mesenchymal cells direct the transformation of the third pharyngeal pouch and surrounding epithelial cells, thereby shaping the thymic primordium (9). In addition, transcriptional and humoral factors produced by mesenchymal cells support thymic epithelial integrity and normal thymic development (10–13). Medullary fibroblasts (mFbs) have also been reported to express TRAs, and impaired maturation of these cells can induce autoimmunity (6, 14).

Mesenchymal stromal subsets also play major roles in thymic egress. Two pathways are particularly well established: the CCL21**–**CCR7 axis and the sphingosine-1-phosphate (S1P)**–**S1PR1 axis (7). Although CCL21 is expressed by mTECs, heparan sulfate on adventitial cells and pericytes surrounding blood vessels captures CCL21 and helps attract mature thymocytes. Disruption of the CCL21**–**CCR7 axis impairs thymic egress and causes defective negative selection and dacryoadenitis (15, 16).

Regulation of the S1P–S1PR1 axis is complex (17, 18). Sphingosine kinases 1 and 2 (*Sphk1* and *Sphk2*) produce S1P. Sphingosine-1-phosphate transporter (*Spns2*) transports the produced S1P to extracellular spaces. Sphingosine-1-phosphate lyase 1 (*Sgpl1*) degrades intracellular S1P to regulate S1P production to appropriate levels. Finally, phospholipid phosphatase 3 (*Plpp3*) on the plasma membrane dephosphorylates extracellular S1P to maintain the gradient. Mesenchymal cells regulate the S1P–S1PR1 axis via expression of at least two genes: *Sphk1* (19) and *Sgpl1* (20). These reports suggest that the production of S1P from mesenchymal cells – not at excessive levels – is required.

While mesenchymal cells contribute to thymic egress, endothelial cells play significant roles in both thymic entry and egress. These specialized endothelial cells are termed thymic portal endothelial cells (TPECs). Entry-associated TPECs promote progenitor entry through adhesion molecules such as P-selectin, VCAM-1, and ICAM-1 (21, 22). On the other hand, egress-associated TPECs regulate thymic egress via S1P: EC-specific deletion of *Spns2* disrupts thymic egress (23–26). Interestingly, endothelial expression of *Plpp3* is likewise required for proper thymic egress (27). This apparent paradox suggests that different endothelial subsets may cooperate to establish the S1P gradient required for efficient egress. However, entry-associated TPECs and egress-associated TPECs remain difficult to distinguish. In a published scRNA-seq dataset, both appear to fall within the same *Selp*^+^*Bst1^+^* cluster, even though BST-1^+^ vessels have been proposed as sites of thymic emigration (28).

These findings led us to hypothesize that mEFAs are not merely structural defects, but specialized medullary regions with distinct stromal organization and function. To test this idea, we sought to identify the differentiation stages of thymocytes and the stromal subsets localized within mEFAs. However, conventional IHC in rat tissue is constrained by the limited availability of rat-reactive antibodies, and many commercially available antibodies do not perform reliably in rat thymic sections. We therefore applied spatial transcriptomics (ST) to obtain an antibody-independent view of mEFA-associated cell populations. At the time this study was conducted, no ST platform optimized specifically for rat tissue was available, and we therefore used a species-independent, reverse-transcription-based platform. Although this approach is applicable to rat samples, transcript capture per cell-equivalent segment is limited, making it difficult to discriminate closely related cell subsets or cellular states from ST data alone. To address this limitation, we generated reference scRNA-seq datasets and used robust cell-type decomposition (RCTD) (29) to infer cellular composition within the ST data. By combining this approach with IHC, we identified accumulation of mature thymocyte subsets, uniquely differentiated mesenchymal subsets, and vECs expressing TPEC-associated markers within mEFAs, thereby providing a transcriptomic characterization of these unusual medullary regions.

## 2 Materials and methods

### 2.1 Animals

Inbred Lewis, F344, DA, and ACI rats, aged 3–8 weeks, were purchased from SLC Co. (Shizuoka, Japan) and maintained under specific pathogen-free conditions. Some animals were kept in the facility to obtain samples from 6-month-old rats. The exact ages and sexes of the animals used in each experiment are indicated in the corresponding figures, figure legends, and source data. All animal procedures were approved by the Dokkyo Medical University Animal Experiment Committee (approval no. 1358), in accordance with the university’s regulations for animal experiments and Japanese Governmental Law (No. 105).

### 2.2 Antibodies

Antibodies used for IHC and immunoprecipitation are listed in **Supplementary Table 1**. Some antibodies were purified from culture supernatants and conjugated in-house.

### 2.3 Immunohistochemistry

Tissues were embedded in Tissue-Tek O.C.T. compound (Sakura Finetek Japan, Tokyo, Japan), snap-frozen, and stored at **−**80 °C. Frozen blocks were sectioned at a thickness of 4 μm. After air drying for 2 h and fixation in acetone, sections were stained as described previously (3, 4). Briefly, acetone-fixed sections were rehydrated in Tris-buffered saline (pH 7.4), and then refixed in 4% paraformaldehyde containing 1% calcium chloride solution. After blocking with Block Ace (KAC, Kyoto, Japan), sections were incubated with primary antibodies for 1 h or overnight. After washing with PBS, sections were further incubated with the appropriate secondary antibodies or streptavidin, and signals were developed using Vector Blue substrate (Vector Laboratories, Newark, CA, USA), the New Fuchsin Substrate System (Agilent, Santa Clara, CA, USA), or 3,3**′**-diaminobenzidine (DAB) substrate (Dojindo Molecular Technologies, Kumamoto, Japan). For multicolor staining, these steps were repeated sequentially.

Photomicrographs were acquired using a BX53 microscope equipped with UPlanFL N objective lenses and a DP27 digital camera (Olympus, Tokyo, Japan). Images were captured with cellSens software (Olympus). Exposure and contrast settings were kept constant within each experiment. For area measurements, montage images of whole thymic sections were generated, and measurements were performed using the cellSens software.

For cross-strain and cross-age analyses, areas containing ED21^+^ thick reticular cells (mTECs) were defined as medullary epithelium-containing areas (mECAs), whereas areas lacking mTECs and filled with delicate type IV collagen fibers were defined as mEFAs (3, 4).

### 2.4 Spatial transcriptomics

A thymus was cut horizontally into three pieces, and the middle portion was embedded in O.C.T. compound in a 1 × 1 cm plastic tray. To maximize the tissue area within the tray, right and left lobes were positioned back to back. A 10-μm-thick section was mounted onto a Chip T (STOmics, Shenzhen, Guangdong, China). The slide was incubated at 37 °C for 5 min and then stored in an airtight container at **−**80 °C. Library preparation and sequencing were outsourced to GENEWIZ (Tokyo, Japan). For cell segmentation, ssDNA staining was performed. The resulting FASTQ files and the image of the ssDNA-stained section were processed using the Stereo-seq Analysis Workflow pipeline (ver. 8.1.3, STOmics), with the mRatBN7.2 genome assembly as a reference. For brief visualization of gene expression, StereoMap (ver. 4.1.2, STOmics) was used.

Among the pipeline outputs, the.h5ad files contain gene-expression matrices, spatial coordinates, and pipeline-generated Leiden cluster assignments. The spatial units in cellbin_1.0.adjusted.h5ad are based on ssDNA-derived cell segmentation, whereas bin20_1.0.h5ad comprises a uniform grid of 20 × 20 DNA-nanoball capture units (approximately 10 µm × 10 µm). These files were imported into the R environment (ver. 4.5.1) using the reticulate (ver. 1.43.0), anndata (ver. 0.8.0), and zellkonverter (ver. 1.18.0) packages. Data from these files were extracted and reconstructed as Seurat (ver. 5.3.0) objects designated CellBin_obj and bin20_obj, respectively. Feature IDs were remapped to gene symbols. Count matrices and coordinates were stored in the obj@assays$RNA@counts and obj@reductions$spatial slots, respectively. The objects were then processed to generate log-normalized expression values (obj@assays$RNA@data). To reduce the object size, z-scored data (obj@assays$RNA@scale.data) were removed.

To identify mECA and mEFA regions, we established mTEC and SP thymocyte marker sets (**Figure 1B**; **Supplementary Table 2**). These marker genes were selected from previous studies (4, 30–33), or identified in our RatThymocyte.obj and RatThymicStroma.obj datasets using the FindAllMarkers() function. These gene sets were applied to the ST datasets using the AddModuleScore() function. The scores were z-scaled and then smoothed by k-nearest-neighbor averaging using the FNN package (ver. 1.1.4.1). In the CellBin_obj, segments within the top 10% of the smoothed SP-thymocyte score and the top 15% of the smoothed mTEC score were defined as mECAs, whereas segments within the top 10% of the smoothed SP-thymocyte score but outside the top 10% of the smoothed mTEC score were defined as mEFAs. In the bin20_obj, the smoothed mTEC score was adjusted to the top 10% based on anatomical concordance with the mECA extent identified in CellBin_obj.

**Figure 1:**
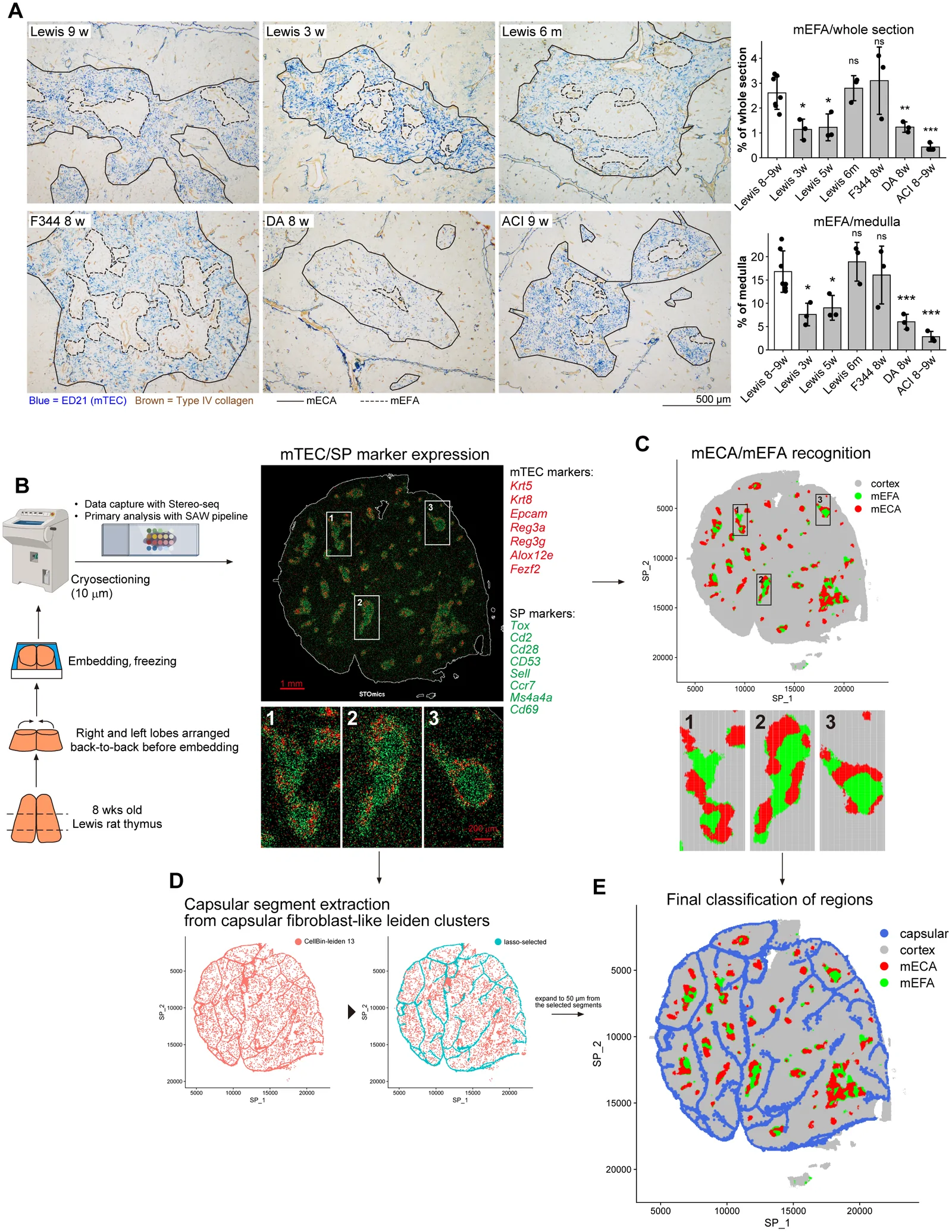
Rat thymic overview and ST data acquisition. **(A)** Representative ED21-stained thymic sections from rats of different ages and strains, with quantification of mEFA areas. Statistical significance was assessed using Student’s t-test. ns: not significant, *p < 0.05, **p < 0.005, ***p < 0.0005. **(B)** Schematic overview of the spatial-transcriptomics workflow and the definitions of mECAs and medullary regions. A male 8-week-old Lewis rat was used. mECAs and medullary regions are depicted as mTEC- and SP thymocyte-associated gene-expressing areas, respectively. Gene expression was visualized using StereoMap. **(C)** mECAs, mEFAs, and cortex regions identified computationally using Seurat in the ST dataset. **(D)** Leiden clusters containing capsular fibroblasts and lasso-selected capsular segments. **(E)** Regions determined in the annotation procedures. **(C–E)** depict Bin20_obj. mEFA, medullary epithelium-free areas; mECA, medullary epithelium-containing areas.

To define capsular regions, capsular segments were manually identified within *Dpp4* and *Pi16*-enriched Leiden clusters, using the lasso_select() function, which we created at line 320 in Stereo-seq_analysis_cellbin.R based on Shiny (ver. 1.11.1) and plotly (ver. 4.11.0). The selected capsular segments were then spatially dilated by 50 μm. Thus, the resulting capsular annotation corresponds to an approximate total capsular width of 100 μm plus the width of the originally selected segments.

### 2.5 Tissue digestion

For scRNA-seq analysis of thymocytes, thymi were minced and incubated at 37 °C for 25 min in an enzyme mixture containing 0.2% collagenase D (Roche Diagnostics, Indianapolis, IN, USA) and 0.01% DNase I (Roche) in Hanks’ balanced salt solution (HBSS) supplemented with 1.25 mM Ca^2+^, 1.45% bovine serum albumin (BSA), and 2% fetal calf serum (FCS). During incubation, the tissues were mechanically dissociated every 5 min by pipetting sequentially with truncated 1000-μL pipet tips, Pasteur pipettes, and heat-tapered tip Pasteur pipettes. After incubation, EDTA was added to a final concentration of 2.5 mM, and the samples were incubated for an additional 5 min. The digested tissue was then filtered through pieces of 50-μm nylon mesh. The resulting cell suspension was centrifuged and resuspended in ACK lysis buffer (145 mM NH_4_Cl, 10 mM KHCO_3_, and 0.1 mM EDTA, pH 7.4). After incubation at 4 °C for 10 min, cells were resuspended in PBS, counted using a hemocytometer, and used for each experiment.

For preparation of thymic stromal cells, thymi were digested using a similar procedure, except that the enzyme mixture contained 0.0125% Liberase TH (Roche) and 0.004% DNase I. After the ACK treatment, cells were resuspended at 1.25 × 10^9^ cells/ml in PBS(−) containing 0.5% BSA and 2 mM EDTA (MACS buffer). To block nonspecific binding, ChromPure^®^ mouse IgG whole molecule (Jackson ImmunoResearch, West Grove, PA, USA) was added at 10 μg/ml. After incubation at 4 °C for 10 min, anti-rat CD45 MicroBeads (Miltenyi Biotec, North Rhine-Westphalia, Germany) were added at 10% of the suspension volume. After incubation at 4 °C for 15 min, the cell suspension was diluted sixfold with MACS buffer and then separated using the Depl05 program on an autoMACS instrument (Miltenyi Biotec). The CD45**^−^** fraction was stained with 7-AAD (IMMUNOSTEP, Salamanca, Spain) and Pacific Blue-conjugated anti-CD45 antibody (BioLegend, San Diego, CA, USA). Viable CD45**^−^** cells were further sorted using a FACSAria III cell sorter (Becton, Dickinson and Company, Franklin Lakes, NJ, USA) and collected in HBSS supplemented with 1.25 mM Ca^2+^, 1.45% BSA, and 2% FCS. The sorted cells were washed three times with PBS(**−**) and then subjected to scRNA-seq analysis.

### 2.6 scRNA-seq and reference construction for RCTD

Thymocyte scRNA-seq and stromal cell scRNA-seq samples were each collected from a distinct animal. Prepared thymocyte and stromal cell suspensions were submitted to the on-site NGS library construction and sequencing service at Azenta Japan Corp. More than 15,000 cells with >90% viability were loaded onto a Chromium Chip G, targeting the recovery of 10,000 cells. Single-cell gel bead-in-emulsions (GEMs) were generated using the Chromium Next GEM Single Cell 3**′** GEM, Library & Gel Bead Kit v3.1 and a Chromium Controller (10x Genomics, Pleasanton, CA, USA). A reverse-transcription reaction was performed within GEMs, during which transcripts from individual cells were labeled with cell-specific barcodes and unique molecular identifiers. The resulting cDNA was amplified and used for 3**′** gene-expression library construction. Libraries were quantified using the Qubit dsDNA Assay (Thermo Fisher Scientific, Waltham, MA, USA) and TapeStation D1000 ScreenTape (Agilent Technologies, Santa Clara, CA, USA), and sequenced on a NovaSeq 6000 platform (Illumina, San Diego, CA, USA), generating approximately 450–500 million paired-end reads per sample. Sequencing reads were obtained with lengths of 28 bp for Read 1 and 90 bp for Read 2.

The resulting FASTQ files were processed using the Cell Ranger pipeline (ver. 8.0.0, 10x Genomics), with the mRatBN7.2 genome assembly as the reference. The output filtered_feature_bc_matrix folders (including expression matrices without non-cell signals) were imported into the R environment and converted into Seurat objects, designated RatThymocyte.obj and RatThymicStroma.obj. The proportion of mitochondrial transcripts was calculated for each cell using PercentageFeatureSet() with the pattern ^mt-. For RatThymocyte.obj, cells with more than 200 detected genes were retained. For RatThymicStroma.obj, cells with 500-7,000 detected genes and less than 5% mitochondrial transcripts were retained. After quality-control filtering, gene-expression counts were log-normalized using NormalizeData() with the default setting (LogNormalize method and scale factor of 10,000). The 2,000 most variable genes were identified using the variance-stabilizing transformation method implemented in FindVariableFeatures(). Expression values for all detected genes were centered and scaled, and principal component analysis (PCA) was performed using the variable genes. PCA dimensionality was evaluated using JackStraw analysis with 100 replicates and elbow plots. A shared nearest-neighbor graph was constructed from the first 10 PCs, corresponding to the default setting of FindNeighbors(), and graph-based clustering was performed using FindClusters() with resolution = 0.5. UMAP embeddings were generated using PCs 1–15 for RatThymocyte.obj and PCs 1–17 for RatThymicStroma.obj. The resulting clusters were annotated based on marker-gene expression as described in the Results.

A published mouse thymocyte dataset was obtained from the Gene Expression Omnibus (GSE186078) (30). Expression matrices from individual animals were imported into R and processed using procedures similar to those used for the rat datasets. To exclude low-quality cells, B6 cells with more than 200 detected genes were retained. AND cells were required to have 1,000–3,500 detected genes and less than 9% mitochondrial transcripts; F5 cells, 1,000–4,000 genes and less than 7% mitochondrial transcripts; MHC-I KO cells, 500–3,500 genes and less than 9% mitochondrial transcripts; and MHC-II KO cells, 1,000–4,000 genes and less than 9% mitochondrial transcripts. For OT-I samples, replicate 1 cells were required to have 1,500–5,000 detected genes and replicate 2 cells 700–3,500 genes, with less than 9% mitochondrial transcripts in both replicates. For OT-II samples, replicate 1 cells were required to have 1,000–4,000 detected genes and replicate 2 cells 500–2,500 genes, again with less than 9% mitochondrial transcripts. After quality-control filtering, cells from all genotypes were merged into a single Seurat object (all_m_thymocyte). RNA counts were log-normalized using NormalizeData() with the LogNormalize method and a scale factor of 10,000. The 2,000 most variable genes were selected using the variance-stabilizing transformation method, scaled, and used for PCA. The layers in the all_m_thymocyte object were then integrated using the IntegrateLayers() function with method = JointPCAIntegration. A shared nearest-neighbor graph and UMAP embedding were calculated using dimensions 1–30. Clustering was performed with the resolution set to 1, generating 33 clusters. We named these clusters following the original study with some modifications (see Results and **Supplementary Figure S1B**). The Pre-Treg cluster was defined manually with the lasso_select() function.

A published mouse thymic stroma dataset, ThymoSight, was obtained from Zenodo (12516405) (34). The file thymosight_cd45neg_TOTAL_mouse.h5ad was imported into R using the zellkonverter package and converted into a Seurat object. The original metadata, clustering, and UMAP coordinates were retained. This object was designated MouseThymicStroma.obj.

To project mouse-derived annotations onto rat cells, gene symbols in the mouse objects were converted to rat orthologs using convert_orthologs() in the orthogene package (ver. 1.14.1), with non121_strategy = “keep_popular” to resolve non-one-to-one mappings without expanding the feature dimension of the expression matrices. Because the function refers to the periodically updated g:Profiler database, all conversions were performed in September 2025, and the resulting ortholog conversion table was saved for reproducibility (**Supplementary Table 3**). The symbol-converted all_m_thymocyte and MouseThymicStroma.obj objects were then integrated with RatThymocyte.obj and RatThymicStroma.obj, respectively, using the IntegrateLayers() function. JointPCAIntegration was used for the thymocyte datasets, whereas CCAIntegration was used for the stromal datasets.

Mouse cluster labels were then projected onto rat cells using a k-nearest-neighbor-based label transfer procedure in the UMAP space of the integrated Seurat objects. The 50 nearest mouse neighbors of each rat cell were identified using the FNN package (ver. 1.1.4.1). Rat-cell annotation was assigned by majority vote among the neighboring mouse cells. A projected label was accepted only when the most frequent donor label accounted for at least 55% of the neighbors; otherwise, the cell was left unlabeled. The projected labels were then transferred back to RatThymocyte.obj and RatThymicStroma.obj, which were subsequently integrated to generate RatThymicRef. After generating RatThymicRef, several minor populations, including NKT, gdT, aaTEC subsets, and mimetic TEC subsets, were excluded to reduce the computational cost of RCTD, and because their representation in the rat dataset was too limited to support reliable annotation. The resulting reduced reference object was named RatThymicRef_l and was used for subsequent RCTD analyses.

The similarities between the original and projected clusters were calculated as Pearson correlation coefficients. The gene expression matrix was extracted from the cross-species integrated scRNA-seq dataset of thymic stroma. Standard deviations were calculated among the projected clusters, and genes were ranked by these deviations. Pearson correlation coefficients between the original mouse clusters and the projected rat clusters were calculated using the expression values of the top 1,000 genes.

### 2.7 RCTD

RCTD was performed using the spacexr package, with reference to a guide (35). The reference object was generated from RatThymicRef_l using the joined raw RNA-count matrix, the consolidated cell-type annotations, and the total RNA UMI count for each reference cell, with the Reference() function. From both CellBin_obj and bin20_obj, the query objects were constructed from the raw RNA-count matrix, the x-y coordinates of each spatial unit, and its total UMI count, using the SpatialRNA() function. RCTD objects were then created using create.RCTD() with the default parameters and analyzed using run.RCTD(doublet_mode = “multi”), allowing more than one candidate cell type to be assigned to individual segments. The ranked candidate cell types, assignment-confidence values, and estimated cell-type weights were extracted from the cell_type_list, conf_list, and sub_weights results, respectively, and added to the metadata of the corresponding spatial transcriptomics objects. Evaluation of the resulting annotations is described in the Results (**Supplementary Figure S3**).

### 2.8 Type 2 cytokine expression and NKT ratios in thymocyte clusters

To compare non-enriched wild-type thymocyte subsets, the r4 and r5 replicates from C57BL/6 mice in the all_m_thymocyte object were extracted because these two batches were not SP-enriched (30). Erythrocytes, B cells, and myeloid cells were further removed from the extracted wild-type mouse dataset and from RatThymocyte.obj. Expression of type 2 cytokines and the ratios of NKT cells were calculated within these datasets.

### 2.9 Gene set enrichment analysis

To perform GSEA on thymocyte-derived transcriptomes across regions, region-grouped expression profiles were extracted from CellBin_obj using the AverageExpression() function. Genes were ranked according to the expression difference between mEFA and mECA. To reduce contamination from stromal transcripts, marker genes for mTECs, TMC3/4, and EC clusters were identified in the RatThymicRef object using FindAllMarkers() and removed from the ranked gene list. GSEA was then performed using clusterProfiler (ver. 4.16.0).

For the reference-dataset GSEA, SP clusters were grouped as mECA-enriched or mEFA-enriched according to their spatial distributions in **Figure 2B**. Grouped expression data were extracted using AverageExpression(), and genes were ranked according to the expression difference between the mEFA- and mECA-enriched groups. GSEA was then performed using the gseGO() function on the resulting ranked list. Under the default settings, GO terms with an adjusted *p*-value of less than 0.05 were considered statistically significant.

**Figure 2:**
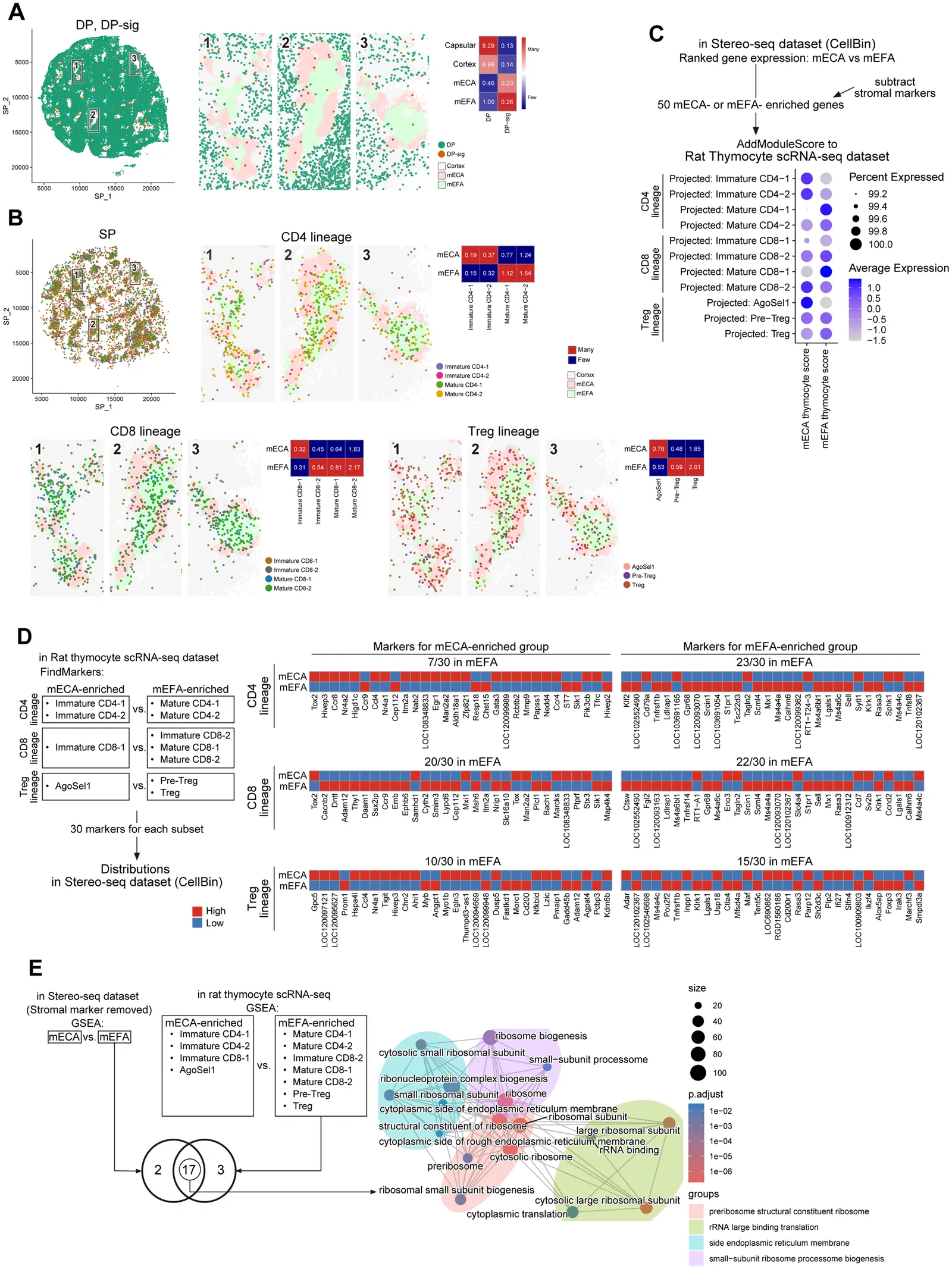
Distribution of thymocyte subsets. **(A)** Distribution of RCTD-predicted segments corresponding to DP and DP-sig subsets in the ST dataset. Numbers in the heatmap indicate the percentage in each region, whereas colors correspond to z-scores across regions. **(B)** Distribution of RCTD-predicted segments corresponding to SP subsets in the ST dataset. Numbers in the heatmap indicate the percentage in each region, whereas colors indicate binarized abundance across mECAs and mEFAs. **(C)** mECA- and mEFA-thymocyte scores within the scRNA-seq reference dataset, based on markers extracted from the ST dataset. **(D)** Regional expression of mECA-enriched and mEFA-enriched SP thymocyte markers within the ST dataset. Markers were extracted from the reference dataset as depicted in the figure. **(E)** Overlap between GSEA results from the ST dataset and the reference scRNA-seq dataset. The shared terms are depicted as an enrichment plot. The size and p.adjust values in the plot were derived from the GSEA of the ST dataset. DP, double-positive thymocyte; DP-sig, DP thymocytes that had received the positive-selection signal; SP, single-positive thymocyte; AgoSel1, agonist-selected subset 1.

Pathways identified in each GSEA result were compared using a Venn diagram.

### 2.10 Lymphotoxin β receptor-dependent genes

A bulk RNA-seq dataset including *Ltbr*-deficient mFb samples was downloaded from GSE147357 (6). Gene counts were averaged within each group and transformed to log2 scale. Genes whose average expression in the *Ltbr*-deficient mFb group was at least 2 log2 units lower than that in the control mFb group were selected. These genes were then filtered against a list of tissue-restricted genes (36). The resulting genes were converted to rat orthologs and considered lymphotoxin β receptor-dependent TRAs in mFbs (**Supplementary Table 4**). This gene set was then applied to RatMouseStroma.obj using the AddModuleScore() function.

### 2.11 RNA velocity analysis

RNA velocity analysis was performed on rat mesenchymal subsets according to a standard protocol (37). A loom file containing spliced and unspliced transcript counts was generated from the rat stromal scRNA-seq data using velocyto (ver. 0.17.17) (38) and merged with the Cell Ranger-filtered gene-expression matrix in a Python environment (ver. 3.6.13). Cells were restricted to the previously annotated mesenchymal populations. Genes with fewer than 30 shared spliced and unspliced counts were filtered out, and the remaining counts were normalized and log-transformed using the scv.pp.filter_and_normalize() function in scVelo (ver. 0.2.4) (39). The 2,000 most variable genes were retained. PCA was performed using 30 components, and a k-nearest-neighbor graph was constructed from these 30 PCs using 30 neighbors. A UMAP embedding was then calculated from this graph. First- and second-order moments were estimated using the same 30-PC, 30-neighbor graph. Gene-specific transcriptional dynamics were recovered using recover_dynamics(), after which RNA velocities were inferred using the dynamical model, a velocity graph was constructed, and latent time was estimated. The dynamics-recovery, velocity-estimation, and velocity-graph steps were run with 24 parallel jobs. Velocity streamlines were projected onto the calculated UMAP embedding.

To characterize transcriptional dynamics, latent time, root-cell probability, end-point probability, and partition-based graph abstraction (PAGA) were calculated. These velocity-derived metadata were exported and integrated back into the Seurat object for downstream visualization and analysis.

### 2.12 Cell-cell interaction analysis

Cell-cell interaction analyses were performed using the CellChat package (ver. 2.2.0) (40). For the rat analysis, gene symbols in the mouse interaction database (CellChatDB.mouse) were converted using the orthogene package. Interactions between clusters were computed using the computeCommunProb() function with the default setting (type = “triMean”). Interactions between stromal subsets and mature SP subsets were extracted from the rat and mouse CellChat result objects using the subsetCommunication() function and then compiled into a combined table. Mouse genes in the table were converted to rat orthologs. No additional filtering was applied during these processes.

### 2.13 Immunoprecipitation and mass spectrometry

A thymus from an 8-week-old Lewis rat was homogenized in 5 mL of ice-cold lysis buffer containing 50 mM Tris-HCl (pH 7.4), 1% NP-40, 125 mM NaCl, 5 mM EDTA, and cOmplete protease inhibitor cocktail (Roche Diagnostics, Indianapolis, IN, USA), using a manual homogenizer (**Supplementary Figure S5B**). The lysate was incubated on ice for 15 min and centrifuged at 1,000 × g for 10 min. The supernatant was further centrifuged at 10,000 × g for 10 min. The resulting supernatant was adjusted to 10 mL and stored at −80 °C until use.

Dynabeads™ M-280 Streptavidin (Invitrogen, Carlsbad, CA, USA) were dispensed into five 5-mL tubes at 100 μL (equivalent to 1 mg of beads) per tube. After adding 1 mL of PBS, the beads were collected using a DynaMag-5 magnetic rack, and the supernatant was removed. This washing process was repeated once, and the beads were resuspended in 100 μL of 0.1% BSA-PBS. Biotin-conjugated antibodies (ED18, ED19, or ED21, 40 μg each; OX3, which recognizes the rat MHC class II molecules RT1-Ba/Bb^u^, as a positive control for the workflow, 20 μg; or isotype-control IgM, 20 μg) were added to the tubes. The suspensions were incubated for 30 min at room temperature with gentle agitation to form bead-antibody complexes. The complexes were washed four times with 0.1% BSA-PBS. Thymic lysate (2 mL) was added to each tube, and the suspensions were incubated for 30 min at 4 °C with gentle agitation. The bead-antibody-antigen complexes were washed three times and resuspended in 100 μL of elution buffer containing 4 M urea and 10 mM dithiothreitol. After incubation at room temperature for 15 min, the beads were collected using the magnetic rack, and the supernatants were recovered. The elution step was repeated once, and the combined eluates were stored at −80 °C until further processing.

Before trypsin digestion, the eluted samples were reduced and alkylated with 30 mM iodoacetamide in 100 mM ammonium bicarbonate. The samples were then adjusted to final concentrations of 1 M urea and 2.5 mM dithiothreitol and digested with sequencing-grade modified trypsin (Promega, Madison, WI, USA) for 16 h at 37 °C. The resulting peptides were desalted and filtered using C18-Tip devices (CPE C-TIP 1000-C18-S2-100, Nikkyo Technos, Tokyo, Japan) before LC-MS/MS analysis. The processed peptides were analyzed by SWATH acquisition on a TripleTOF 6600 system (SCIEX, Marlborough, MA, USA). The acquired data were then processed using DIA-NN (ver. 2.2.0 Academia) (41). A rat protein sequence database was prepared from the UniProtKB *Rattus norvegicus* canonical FASTA file. DIA-NN was run in library-free mode. A predicted spectral library was generated from the FASTA file and used for the subsequent DIA search. The following precursor generation settings were used: protease, Trypsin/P; missed cleavages, 1; peptide length, 7–30 amino acids; precursor charge range, 1–4; precursor m/z range, 300–1800; and fragment ion m/z range, 200–1800. N-terminal methionine excision was enabled. Cysteine carbamidomethylation was set as a fixed modification. Variable modifications were not included in the first-pass analysis. The output was filtered at 1% FDR. Match-between-runs was not used for the final quantitative comparison.

Molecular weights of proteins were automatically obtained from UniProt and added to the DIA-NN output (**Supplementary Table 5**) using an R script (add_uniprot_mw.R). In the DIA-NN output, the antibody-specific enrichment was calculated as:


$$Ab_{enrich}=\log_{2}\left(\frac{I_{Ab}+1}{I_{IgM}+1}\right)$$

where *I* denotes the DIA-NN-derived protein-group quantity in each immunoprecipitation. The ED21-specificity score was defined as:


$$ED21_{spec}=ED21_{enrich}-\max\left(ED18_{enrich},ED19_{enrich}\right)$$

Proteins satisfying the following criteria were considered candidate ED21 antigens and subjected to further investigation: *ED21_enrich_* > 0, *ED21_spec_* > 0.3, and 45< MW (kDa)< 55 (**Supplementary Figure S5C**).

## 3 Results

### 3.1 Rat thymic overview and ST data acquisition

We used the ED21 antibody to depict medullary areas, as this antibody specifically binds to rat mTECs (4) and its antigens are presumably keratins 14 and 17 (discussed later). As we previously reported, the thymic medulla of 8–9-week-old Lewis rats contains mTEC-deficient mEFAs containing delicate type IV collagen fibers (3, 4) (**Figure 1A**). We further assessed age- and strain-dependent variation in mEFA extent. In Lewis rats, mEFAs were limited at 3 and 5 weeks of age, expanded by 8–9 weeks, and remained comparably extensive at 6 months. At 8–9 weeks, DA and ACI rats exhibited smaller mEFAs than Lewis rats, whereas F344 rats showed a similar extent.

To spatially define these regions, we performed Stereo-seq on a thymic section from an 8-week-old Lewis rat and processed the data using the Stereo-seq Analysis Workflow pipeline (**Figure 1B**). This yielded several h5ad-formatted spatial transcriptomics datasets, of which we used two: an ssDNA-based cell-segmented dataset (CellBin) and a uniformly segmented dataset at 20-bin (10 μm) resolution (Bin20). We annotated medullary compartments based on marker expression, defining SP-marker^hi^ mTEC-marker^hi^ regions as medullary epithelium-containing areas (mECAs) and SP-marker^hi^ mTEC-marker^low^ regions as mEFAs. These markers were selected from previous studies (4, 30–33) or identified as specific markers in our rat scRNA-seq datasets (**Supplementary Table 2**). The combined area of these two regions was considered the medulla.

The CellBin and Bin20 datasets were imported into Seurat, where mTEC and SP marker scores were calculated and smoothed by k-nearest-neighbor averaging to facilitate recognition of mECAs and mEFAs (**Figure 1C**). Capsular regions, including both the outer capsule and interlobular capsular regions, were identified using capsular fibroblast-like Leiden clusters (**Figure 1D**). Capsular segments were manually selected and then expanded by 50 μm to generate capsular-region annotations. These annotations were then combined to generate the final spatial map used for downstream analyses (**Figure 1E**).

### 3.2 Generation of reference scRNA-seq datasets and cell-type identification

As references for cell type identification in the ST dataset, we performed scRNA-seq for rat thymocytes (**Supplementary Figure S1A**) and thymic stroma (**Supplementary Figure S2A**). However, the numbers of cells analyzed were too small to identify clusters for each T-cell developmental pathway or mTEC subtype. Therefore, we further deployed published mouse datasets for projection mapping.

We obtained an scRNA-seq dataset of 8.5 × 10^3^ thymocytes from a Lewis rat (**Supplementary Figure S1A**). The dataset was projected onto UMAP space, and clusters were identified with typical markers for developmental stages (30). As a mouse thymocyte reference, we used a dataset comprising 7.7 × 10^4^ cells from CD4-fated, CD8-fated, and wild-type animals (30). We performed clustering and annotation following the original study, with several modifications (**Supplementary Figure S1B**). First, for exploratory spatial analysis, the SP subsets were divided into more detailed subsets (for example, immature CD4SP and mature CD4SP clusters in the original reference were divided into immature CD4SP-1, immature CD4SP-2, mature CD4SP-1, and mature CD4SP-2), based on the directions of development and the representative transcription factors *Zbtb7b* and *Runx3* (**Supplementary Figure S1C**). Second, the *Ikzf2*^+^*Bcl2l11*^+^*Nr4a1*^+^ cluster, designated Neg. Sel (1). in the original paper, was divided into agonist-selected (AgoSel) 1 and 2, as AgoSel1 expressed markers of agonist-selected Treg precursors, including *Nr4a1*, *Nr4a3*, *Trib1*, *Cd69*, *Nfkb1*, and *Nfkbia* (42, 43) (**Supplementary Figure S1D**). In contrast, AgoSel2 expressed genes characteristic of gut intraepithelial lymphocyte precursors, including *Pdcd1*, *Nrgn*, and *Cd160* (43). Third, the *Tnfrsf4*^+^*Tnfrsf18*^+^ Neg. Sel (2). cluster in the original reference was renamed Pre-Treg based on its proximity to the Treg cluster. The rat and mouse thymocyte datasets were integrated using the JointPCAIntegration algorithm, and the mouse clustering was projected onto the rat cells (**Supplementary Figure S1E**).

For rat thymic stromal scRNA-seq, we magnetically sorted CD45**^-^** cells from the thymus of a Lewis rat, performed sequencing, and mapped the cells onto the UMAP space (**Supplementary Figure S2A**). Capsular fibroblasts (capFb), vascular smooth muscle cells and pericytes (vSMC/PC), and endothelial cells (Endo) were identified based on previous rat and mouse literature (7, 8, 44–46). TECs were identified with conventional rat TEC markers (3, 4, 33). Clusters located between capFb and vSMC/PC were designated thymic mesenchymal cells (TMC). The TMC, Endo, and TEC clusters were temporarily labeled TMC1**–**4, Endo-1/2, and TEC-a**–**e, respectively. As a mouse thymic stroma reference, we used a dataset comprising 3.0 × 10^5^ cells from animals of various ages and genotypes and retained its original clustering and UMAP coordinates (34). The rat and mouse stromal datasets were integrated using the CCAIntegration algorithm, and mouse cluster identities were projected onto the rat cells (**Supplementary Figure S2B**). However, capFb, vSMC/PC, and TMC1–4 clusters were retained with their labels to enable detailed analyses of spatial allocation and interspecies differences (**Figures 3**, **4**). The similarities between the original mouse clusters and the projected rat clusters were confirmed with Pearson correlation (**Supplementary Figure S2C**).

**Figure 3:**
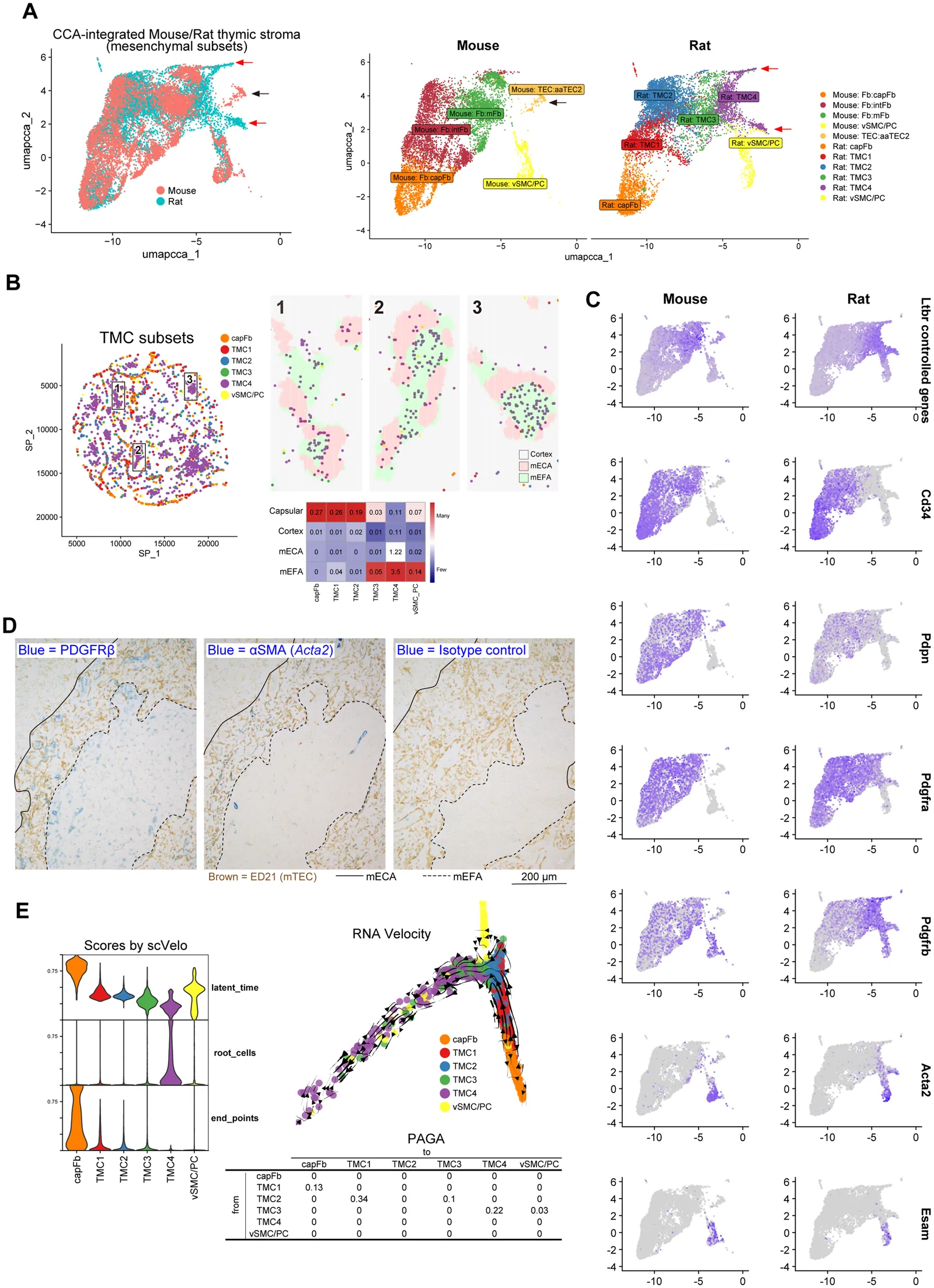
Characterization of mesenchymal subsets and their distribution in the ST dataset. **(A)** Mouse and rat mesenchymal subsets within the cross-species integrated scRNA-seq datasets of thymic stroma. The integration and UMAP projection are based on the CCAIntegration algorithm. Black and red arrows indicate mouse- and rat-unique cells, respectively. **(B)** Distribution of RCTD-predicted segments corresponding to mesenchymal subsets in the ST dataset. Numbers in the heatmap indicate the percentage in each region, whereas colors correspond to z-scores across regions. **(C)** Expression of representative marker genes in mouse and rat mesenchymal subsets. The *Ltbr*-controlled genes are provided in **Supplementary Table 4**. **(D)** Images of sections immunohistochemically stained with anti-PDGFRβ or anti-αSMA. A thymus from an 8-week-old male Lewis rat was used. **(E)** RNA velocity analysis of rat mesenchymal subsets. Violin plots of latent time, root-cell probability, end-point probability, a UMAP plot of RNA velocity, and a table of partition-based graph abstraction (PAGA) scores are presented. capFb, capsular fibroblast; intFb, intermediate fibroblast; mFb, medullary fibroblast; vSMC/PC, vascular smooth muscle cell/pericyte; aaTEC2, aging-associated TEC 2; TMC, thymic mesenchymal cell.

**Figure 4:**
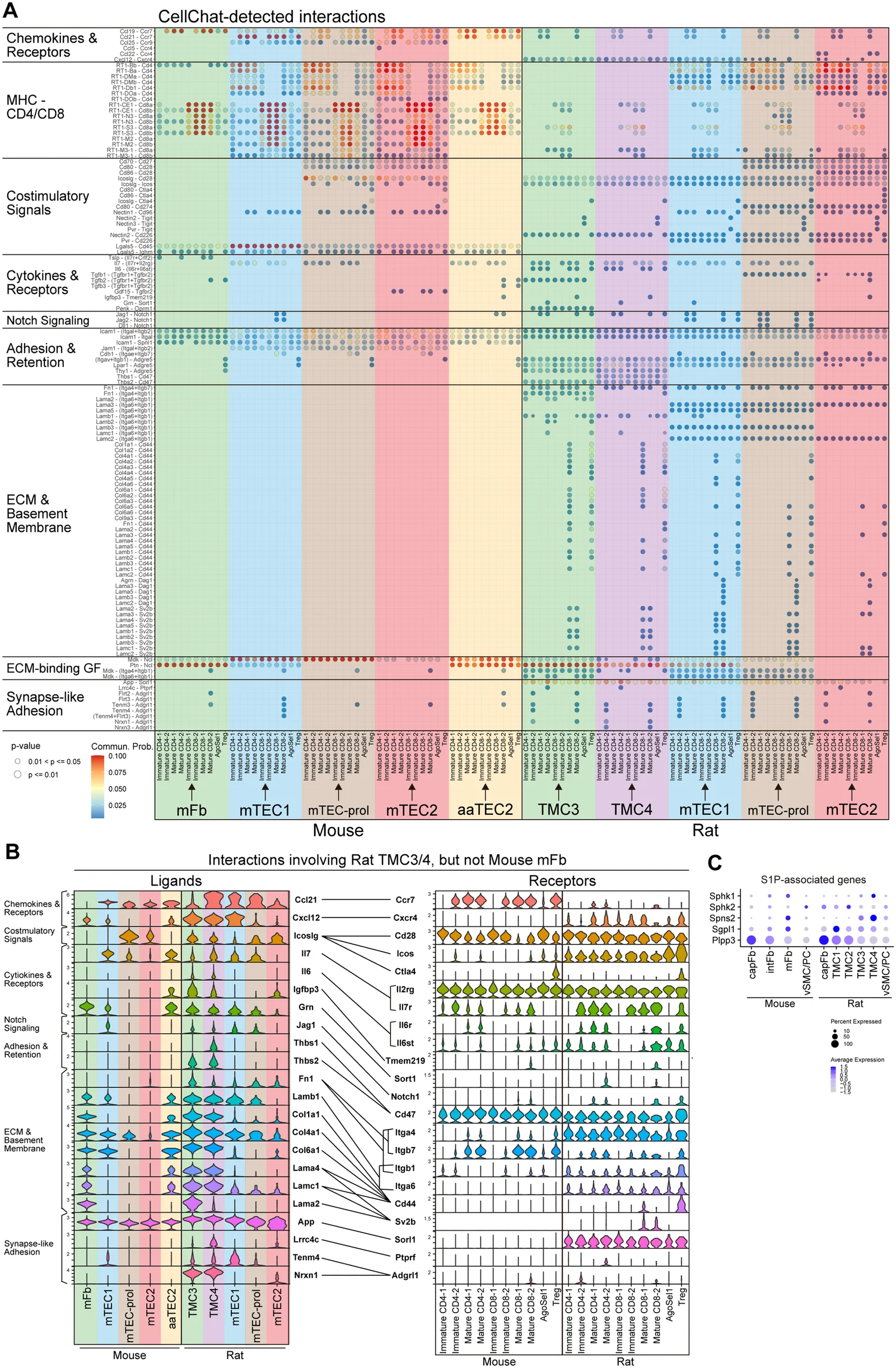
CellChat-detected interactions between thymic stromal subsets and SP thymocytes, and S1P-associated gene expression. **(A)** CellChat-detected interactions between thymic stroma and SP thymocytes. Results from mouse and rat datasets are combined and displayed in a single table. **(B)** Violin plots of ligand and receptor genes underlying predicted rat TMC3/4–SP interactions for which the corresponding mouse mFb–SP interactions were not detected. **(C)** Expression of S1P-associated genes in mouse and rat mesenchymal subsets. In panels A and B, mouse gene symbols are converted to their rat orthologs. In **(C)** expression values are scaled separately within each species. mTEC1, CCL21^+^MHCII^low^AIRE^-^ mTEC; mTEC-prol, proliferating mTEC; mTEC2, CCL21^-^MHCII^hi^AIRE^+^ mTEC.

The rat thymocyte and stromal objects with projected clusters were consolidated into a single reference object (**Supplementary Figure S3A**). Some unreliable clusters (intCD4, mature cycling T, NKT, gdT, MEC, nmSC, TEPC, aaTEC1, aaTEC2, mimetic TEC subsets, and fetus-derived cells) were removed due to their scarcity and/or their distance from the original mouse clusters. Based on this refined reference object, cell types were assigned to the segments in the CellBin and Bin20 ST datasets using RCTD.

In both the CellBin and Bin20 datasets, segments predicted as thymocyte lineages were more numerous than those predicted as stromal lineages when only the top-1 RCTD prediction was considered (**Supplementary Figure S3B**). However, thymocytes showed top2/top1 ratios (defined as the number of segments assigned to each cluster as the second RCTD candidate divided by the number assigned as the first candidate) below 1.00, whereas many stromal lineages showed ratios above 1.00. These observations suggested that stromal transcripts were often captured within segments whose major transcriptomic component was thymocytic. Indeed, mTEC-associated genes (including *Cd74*, keratins, and MHC class II genes), TMC-associated genes (including *C7*, *C4a*, and *S100a5*), and EC-associated genes (*Cst3* and *Timp3*) showed distinct regional enrichment: the mTEC- and EC-associated genes were enriched in mECA-localized segments, whereas the TMC-associated genes were enriched in mEFA-localized segments (**Supplementary Figure S3C**; the full gene list is provided in **Supplementary Table 6**).

We interpreted this pattern as reflecting unavoidable transcriptomic mixing between thymocytes and adjacent stromal cells. In the CellBin dataset, ssDNA-based segmentation may not fully capture thin TEC or fibroblast protrusions, nor the flattened morphology of endothelial cells, causing transcripts from these cells to be assigned to neighboring thymocyte-dominant segments (**Supplementary Figure S3D**). In the Bin20 dataset, transcriptomic mixing is expected by design because each segment represents a fixed 10-μm spatial bin. However, this uniform segmentation was expected to make stromal transcript contamination less dependent on cell shape and therefore more suitable for comparing the spatial distribution of stromal subsets.

Based on this reasoning, we used the CellBin dataset for thymocyte localization analyses and considered only top-1 RCTD predictions. For analyses of stromal subsets, we used the Bin20 dataset. For clusters with moderate top2/top1 ratios, only top-1 predictions were used. For clusters with particularly high top2/top1 ratios (> 4.2), top-2 predictions were also included because restricting the analysis to top-1 predictions would leave too few segments for stable spatial analysis. These clusters were capFb, TMC1, TMC2, TMC4, early Pr (early TEC progenitor), cTEC, and capEC (**Table 1**).

**Table 1:** RCTD predictions in the ST dataset included in analyses.

			Clusters	
dataset	RCTD prediction	Thymocyte lineage	TMC3, vSMC/PC, mTEC1, mTEC-prol, mTEC2, aEC, vEC	capFb, TMC1, TMC2, TMC4, early Pr, cTEC, capEC
CellBin	Top 1	analyzed	not analyzed	not analyzed
	Top 2	not analyzed	not analyzed	not analyzed
Bin20	Top 1	not analyzed	analyzed	analyzed
	Top 2	not analyzed	not analyzed	analyzed

### 3.3 TEC distributions and the iNKT–type 2 cytokine–TEC axis showed features shared between the analyzed rat and mouse datasets

The distributions of TEC subsets in the ST dataset were visualized, demonstrating the expected localization of cTECs in capsular regions and the cortex and of mTECs in mECAs (**Supplementary Figure S4A**). The early Pr (early TEC progenitor) subset was enriched in the capsular regions and mEFAs.

In mice, both CD1d-deficient mice, which lack iNKT cells, and IL-4Rα-deficient mice exhibit mEFA-like structures, suggesting that disruption of interactions between iNKT cell-derived type 2 cytokines and IL-4 receptors on mTECs can promote the formation of mEFA-like regions (47). We therefore examined type 2 cytokine expression and NKT-cell abundance in the thymocyte scRNA-seq datasets (**Supplementary Figure S4B**). *Il4* expression was concentrated in the NKT subset. However, NKT cells were markedly less abundant in the rat dataset than in the mouse dataset. Only 18 rat NKT cells were detected; therefore, the frequency of *Il4*-expressing cells within the rat NKT subset could not be reliably estimated.

We further examined IL-4 receptor components and downstream signaling genes in TEC subsets (**Supplementary Figure S4C**). *Il4ra*, *Il2rg*, *Il13ra1*, *Jak1*, *Jak2*, *Jak3*, *Tyk2*, *Irs1*, *Irs2*, and *Stat6* were expressed in TEC clusters, especially in mTEC2 clusters, in both mouse and rat. Thus, the components of both type I (*Il4ra* + *Il2rg*) and type II (*Il4ra* + *Il13ra1*) IL-4 receptor complexes, together with their downstream signaling machinery, were conserved in rat TECs.

These findings suggest that reduced availability of iNKT-derived type 2 cytokines, rather than loss of the corresponding receptor machinery in TECs, may contribute to mEFA formation in the rat thymus.

### 3.4 Mature SP thymocytes were preferentially localized in mEFAs

The ratios of RCTD-predicted cell types in each area (capsular area, cortex, mECA, and mEFA) were calculated. As is well established, the double-positive (DP) thymocytes were predominantly distributed in capsular areas and the cortex, whereas DP-sig (DP thymocytes that had received the positive-selection signal) appeared to have initiated migration toward the medulla (**Figure 2A**).

Within the SP thymocyte clusters, immature CD4-1, immature CD4-2, immature CD8-1, and AgoSel1 clusters were distributed predominantly in mECAs, whereas mature CD4-1, mature CD4-2, immature CD8-2, mature CD8-1, mature CD8-2, Pre-Treg, and Treg subsets exhibited mEFA-skewed localization (**Figure 2B**). In particular, Treg enrichment in mEFAs was consistent with our previous study (3).

To further examine these localization patterns, we extracted mECA- or mEFA-enriched gene sets from the ST dataset and applied them to the rat thymocyte scRNA-seq dataset using the AddModuleScore() function (**Figure 2C**). Consistent with **Figure 2B**, immature CD4-1, immature CD4-2, immature CD8-1, and AgoSel1 clusters exhibited higher mECA-thymocyte scores, whereas mature CD4-1, mature CD4-2, mature CD8-1, Pre-Treg, and Treg subsets exhibited higher mEFA-thymocyte scores. Although the mature CD8–2 cluster is classified as a mature subset, it displayed a high mECA-thymocyte score. Given its proximity to the mature cycling T cells (**Supplementary Figure S1B**) and lower expression of *S1pr1* and *Sell* (**Supplementary Figure S1D**), the mature CD8–2 cluster might not be a typical egressing subset.

We also tested gene set application from scRNA-seq to ST. We divided the clusters within each lineage into mECA- or mEFA-enriched groups according to their distributions in **Figure 2B** and then extracted 30 marker genes for each group. The expression of these marker sets was examined in the ST dataset (**Figure 2D**). In all lineages, marker sets for mEFA-enriched groups showed greater enrichment in mEFAs than marker sets for mECA-enriched groups.

Finally, to corroborate this tendency of relatively mature phenotypes to localize to mEFAs, we compared two GSEA results: mECA versus mEFA in the ST dataset and mECA-enriched versus mEFA-enriched groups of clusters in the rat thymocyte scRNA-seq dataset (**Figure 2E**). Seventeen of the 22 significantly enriched GO terms were shared, most of which were ribosome-associated. This concordance is interpreted as confirmation of the preferential localization of relatively mature clusters in mEFAs. The list of all detected GO terms is provided as **Supplementary Table 7**.

Of note, because RCTD-predicted thymocyte segments included stroma-derived genes (**Supplementary Figure S3C**), we subtracted these genes from the region-enriched gene sets (**Figure 2C**) and the GSEA (**Figure 2E**).

### 3.5 Rat TMC3/4 subsets were localized in mEFAs and corresponded to mouse medullary fibroblasts but showed unique marker-expression patterns

To examine how rat and mouse thymic mesenchymal clusters correspond to each other, the CCA-integrated mesenchymal subsets were compared (**Figure 3A**). Consistent with the Pearson correlation analysis (**Supplementary Figure S2C**), TMC1/2 and TMC3/4 clusters were considered to be counterparts of mouse intermediate fibroblasts (intFb) and medullary fibroblasts (mFb), respectively. However, a fraction of cells in the rat TMC4 cluster did not overlap with the mouse mFb cluster, suggesting that TMC4 has a partially distinct gene-expression pattern. The mouse aging-associated TEC 2 (aaTEC2) cluster, which exhibits an intermediate gene expression profile between fibroblast and TEC clusters (34) (**Supplementary Figure S2C**), was located near, but did not overlap with, the rat TMC4 cluster.

Next, we investigated the localization of rat mesenchymal subsets in the Stereo-seq dataset (**Figure 3B**). capFb and TMC1/2 clusters were mainly localized in capsular regions, whereas TMC3/4 and vSMC/PC clusters were enriched in mEFAs. Lymphotoxin β receptor-dependent genes in mFb are thought to function as mFb-derived TRAs (6, 8, 14); they were expressed in TMC3/4 (**Figure 3C**; the list of genes is provided in **Supplementary Table 4**). At the same time, several fibroblast marker molecules showed species-specific expression patterns. Although *Cd34* and *Pdgfrb* were expressed in both intFb and mFb clusters in mice, *Cd34* expression was absent from rat TMC3/4, whereas *Pdgfrb* expression was largely restricted to TMC3/4. *Acta2* and *Esam* were specifically expressed in vSMC/PC in both mouse and rat. Based on these marker specificities, we performed immunohistochemistry to further confirm the localization of the TMC clusters (**Figure 3D**). PDGFRβ protein was detected mainly in mEFAs, consistent with the localization of TMC3/4. αSMA (*Acta2*) staining was observed around blood vessels within mEFAs, corresponding to vSMC/PC. αSMA expression was also detected in a subset of mTECs, as suggested by the initial analysis (**Supplementary Figure S2A**).

Finally, to investigate the transcriptional states of TMC clusters, we performed RNA velocity analysis (**Figure 3E**). The TMC4 cluster exhibited the highest root cell probability, while the capFb cluster exhibited the highest latent time and end-point probability. Although partition-based graph abstraction (PAGA) analysis suggested weak transition pathways, velocity streamlines projected onto the UMAP showed bidirectional flows between TMC4 and TMC2/3, indicating heterogeneous transcriptional dynamics among these subsets. Taken together, these analyses suggest that capFb represents a predicted endpoint, whereas TMC4 occupies an upstream transcriptional position. However, the velocity signals among TMC2, TMC3, and TMC4 were mixed, preventing a clear linear ordering of these subsets.

### 3.6 Predicted interactions and S1P-associated expression patterns in rat TMC3/4 subsets

To predict the roles of rat TMC subsets in T cell development, we performed cell**–**cell interaction analysis using CellChat (40). All detected interactions of mFb/TMC, aaTEC2, and mTEC subsets with SP thymocyte subsets are displayed (**Figure 4A**). The detected interactions were grouped into the categories Chemokines & Receptors, MHC – CD4/CD8, Costimulatory Signals, Cytokines & Receptors, Notch Signaling, Adhesion & Retention, ECM & Basement Membrane, ECM-binding growth factors, and Synapse-like Adhesion. We identified a substantial number of TMC3/4**–**SP thymocyte interactions that were not detected in the mouse dataset. Notably, the ECM & Basement Membrane category contained the largest number of these interactions. A complete list of detected interactions is provided in **Supplementary Table 8**. Interactions detected only in the rat analysis were also not detected in the mouse dataset before ortholog conversion, indicating that their non-detection in the mouse dataset was not introduced by the conversion procedure.

Next, to identify the gene-expression patterns underlying these dataset-specific differences, we extracted predicted TMC3/4–SP interactions for which the corresponding mouse mFb–SP interactions were not detected. Expression of the corresponding ligand and receptor genes was then visualized using violin plots (**Figure 4B**). Some ligand genes, such as *Ccl21*, *Icoslg*, *Il7*, and *Thbs1/2*, were expressed in rat TMC3/4 but absent from mouse mFbs. In other cases, ligands were expressed in both rat TMC3/4 and mouse mFbs, whereas the corresponding receptors, such as *Cxcr4*, *Sort1*, *Itga6*, *Cd44*, *Sv2b*, and *Sorl1*, were expressed in rat SP subsets but absent from mouse SP subsets. Notably, among these receptors, *Itga6*, *Cd44*, and *Sv2b*, which bind to laminins and collagens, accounted for the large number of mesenchyme-SP interactions detected only in the rat dataset in the ECM & Basement Membrane category.

Interestingly, *Ccl21* and *Il7* were expressed not only in rat TMC3/4 but also in the mouse aaTEC2 subset. This observation raises the possibility that some functions of rat TMC3/4 may be performed by aaTEC2 cells in mice.

As stated in the Introduction, the S1P–S1PR1 axis is essential for thymic egress. Therefore, we also examined S1P-associated gene expression in mesenchymal clusters (**Figure 4C**). In both the rat and mouse datasets, *Sphk1* and *Spns2* were preferentially expressed in TMC4/mFb clusters, whereas *Plpp3* was expressed in capFb clusters. These differences may contribute to spatial compartmentalization of S1P-metabolic programs. Interestingly, *Sgpl1* was preferentially expressed in the rat TMC1 cluster and the mouse mFb cluster. Thus, its localization differed between the rat and mouse datasets, which could alter spatial S1P regulation in the rat thymus, possibly due to extensive mEFAs.

### 3.7 mEFAs contained thymic portal endothelial cells

In the current consensus, murine ECs are largely divided into capillary EC (capEC), venous EC (vEC), and arterial EC (aEC) (34, 48). Our thymic stromal scRNA-seq dataset also included these three phenotypes (**Figure 5A**). The lymphatic EC marker *Lyve1* was expressed in only a small number of cells and was therefore considered negligible (data not shown).

**Figure 5:**
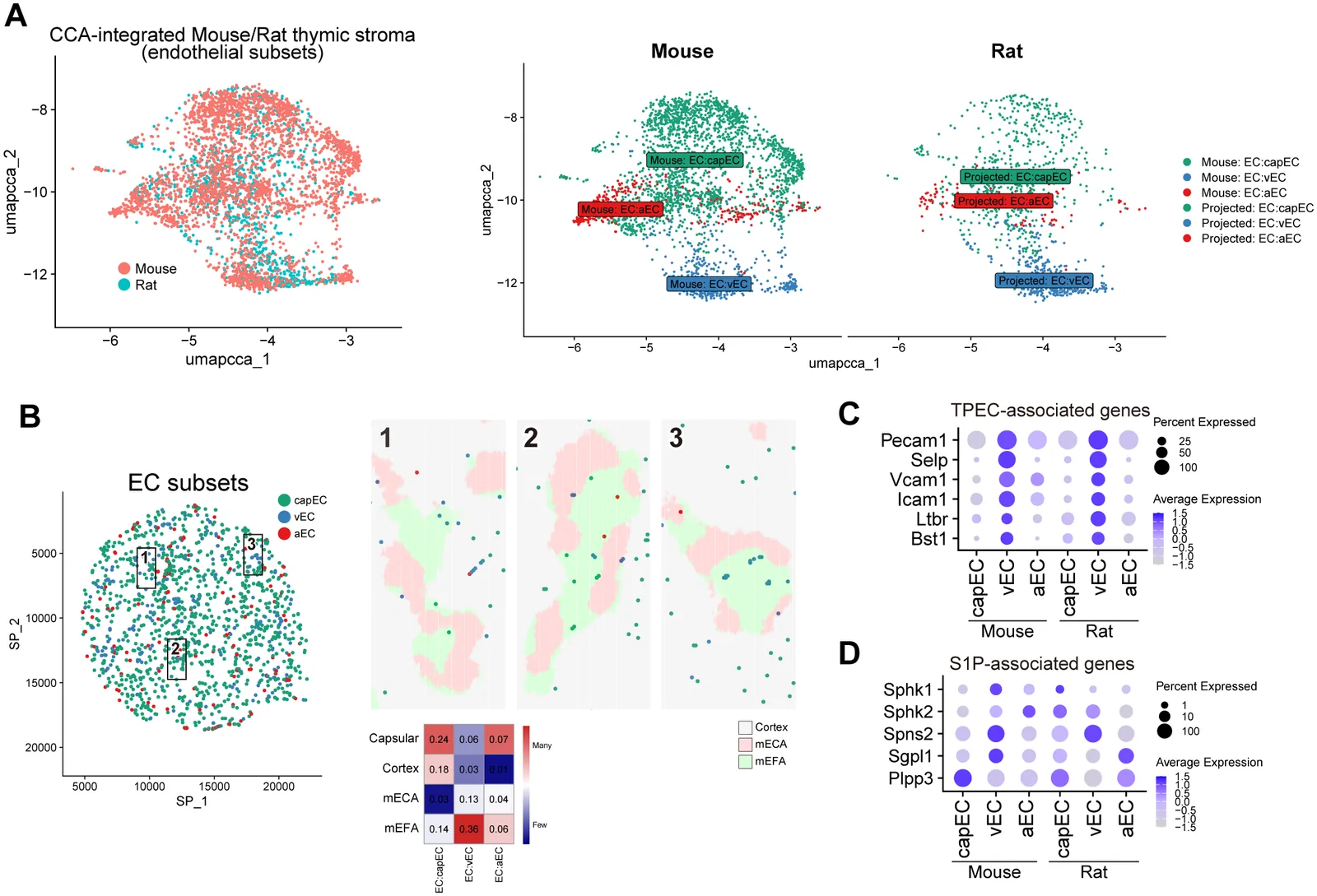
Characterization of endothelial subsets and their distribution in the ST dataset. **(A)** Mouse and rat endothelial subsets within the cross-species integrated scRNA-seq datasets of thymic stroma. The integration and UMAP projection are based on the CCAIntegration algorithm. **(B)** Distribution of RCTD-predicted segments corresponding to endothelial subsets in the ST dataset. Numbers in the heatmap indicate the percentage in each region, whereas colors correspond to z-scores across regions. **(C)** Expression of TPEC-associated genes in mouse and rat endothelial subsets. **(D)** Expression of S1P-associated genes in mouse and rat endothelial subsets. In panels C and D, expression values are scaled separately within each species. capEC, capillary endothelial cell; vEC, venous endothelial cell; aEC, arterial endothelial cell.

RCTD analysis of the ST dataset revealed that capECs were mainly distributed in capsular areas, whereas vECs were distributed predominantly in mEFAs (**Figure 5B**).

TPECs express P-selectin (*Selp*), ICAM-1, and VCAM-1, and require signaling via LTβR to exert full potency (7, 21, 22, 49). Therefore, we investigated expression of these transcripts within EC clusters. All of these genes were concentrated in the vEC cluster (**Figure 5C**). Although TPECs have been reported as entry sites for T cell progenitors, it is also known that BST-1^+^ TPECs function as exit sites for mature thymocytes (28). Consistent with this, *Bst1* was also preferentially expressed in the vEC cluster. However, it was difficult to distinguish entry- and egress-associated TPECs in the scRNA-seq dataset.

Because endothelial cells also regulate the S1P–S1PR1 axis, we examined S1P-associated genes in EC clusters (**Figure 5D**). *Spns2* was preferentially expressed in vECs, whereas *Plpp3* was predominantly expressed in capECs. Thus, S1P export- and degradation-associated genes were segregated between distinct EC subsets, with the export-associated program concentrated in vECs, which were abundant in the mEFAs.

### 3.8 Identification of candidate ED21 target proteins

In our previous study, we showed that ED21 preferentially stains MHCII^low^AIRE**^-^** mTECs, and that the apparent molecular weight of the ED21-reactive protein is approximately 50 kDa (4) (**Supplementary Figure S5A**). In the present scRNA-seq reference dataset, MHC class II- and *Aire*-related genes were preferentially expressed in the mTEC2 cluster, suggesting that the ED21-reactive antigen is more strongly associated with mTEC1-like cells than with mTEC2 cells.

To identify candidate ED21 target proteins, rat thymic lysates were immunoprecipitated with biotin-conjugated ED18, ED19, ED21, OX3, or isotype-control IgM antibodies, followed by DIA-MS analysis of the precipitated samples (**Supplementary Figure S5B**). The complete list of detected protein groups is provided in **Supplementary Table 5**. As a positive control, OX3 immunoprecipitation resulted in the detection of RT1-Ba and RT1-Bb protein groups, confirming that our procedure could recover known antibody targets. ED21-specific, molecular-weight-matched protein groups were selected as candidate ED21 antigens and examined in the reference scRNA-seq dataset (**Supplementary Figure S5C**). Among the candidates, *Krt14*, *Idh1*, *Krt42*, and *Krt17* showed TEC-associated expression. Of these, *Krt14* and *Krt17* were less abundant in the mTEC2 cluster, consistent with the ED21 staining pattern. The spatial expression of these genes was further examined in the ST dataset (**Supplementary Figure S5D**). *Idh1* and *Krt42* did not reproduce the ED21-like spatial pattern, whereas *Krt14* and *Krt17* were concentrated in mECAs. In addition, *Krt14* was detected along capsular regions, corresponding to ED21 staining observed along the capsule by IHC (**Supplementary Figure S5E**), in concordance with the early Pr cluster (**Supplementary Figure S4A**) and human epithelial stem cells (50).

Because ED21 also stains mouse thymic sections, we next surveyed the expression of *Krt14*, *Krt17*, and related paralogs in the rat and mouse reference datasets (**Supplementary Figure S5F**). *Krt14* and *Krt17* were expressed in both mouse and rat TEC clusters. Although *Krt13* and *Krt15* were also expressed in rat TECs, they were not detected among ED21-enriched immunoprecipitated proteins (**Supplementary Table 5**), making them less likely candidates under the present experimental conditions.

These findings suggest that ED21 may recognize a keratin-family epitope represented by keratin 14 and keratin 17 in rat and mouse thymi. Sequence alignment revealed highly conserved regions among these proteins, providing a possible explanation for this cross-reactivity (**Supplementary Figure S5G**).

## 4 Discussion

In this study, by combining scRNA-seq and spatial transcriptomics with IHC, we showed that rat thymic mEFAs are enriched in mature SP thymocyte subsets, mesenchymal subsets similar to mouse medullary fibroblasts but with distinct gene-expression profiles and predicted interactions, and thymic portal endothelial cells associated with thymic egress.

A fundamental question is why mEFAs develop in rat thymi. In mice, both CD1d-deficient and IL-4Rα-deficient models exhibit mEFA-like structures together with reduced thymic egress, suggesting that interactions between iNKT cell-derived type 2 cytokines and IL-4 receptors on mTECs are required for proper medullary organization and function (47). Consistent with this model, NKT cells were markedly less abundant in the Lewis rat thymocyte scRNA-seq dataset than in the C57BL/6 dataset (**Supplementary Figure S4B**). The paucity of thymic iNKT cells in Lewis rats has also been demonstrated independently by CD1d dimer staining (51). In contrast, expression of IL-4 receptor components and downstream signaling molecules in mTECs was broadly conserved between mouse and rat (**Supplementary Figure S4C**), suggesting that the molecular machinery required for type 2 cytokine responsiveness is retained in rat mTECs. Notably, mTEC2 showed the highest expression of IL-4 receptor components among TEC subsets. Because mTEC2 also expresses *Aire* (**Supplementary Figure S5A**) (34, 52), this finding is consistent with the reported iNKT-dependent emergence of an AIRE^+^ TEC population in reaggregated thymic organ cultures (53). Together, these observations support a model in which reduced availability of iNKT-derived type 2 cytokines, rather than impaired cytokine responsiveness of mTECs, contributes to mEFA formation in Lewis rats.

However, disruption of this iNKT–type 2 cytokine–TEC axis does not necessarily result in overt immunodeficiency. Despite these thymic abnormalities, IL-4Rα- and CD1d-deficient mice do not exhibit profound peripheral T-cell lymphopenia (47, 54), and Lewis rats likewise do not spontaneously develop immune-mediated disease under normal conditions. Nevertheless, Lewis rats are highly susceptible to multiple experimentally induced autoimmune diseases, including adjuvant arthritis (55), experimental autoimmune encephalomyelitis (56), uveitis (57), and experimental autoimmune neuritis (58). One possibility is that reduced iNKT-derived type 2 cytokine signaling alters mTEC organization or function sufficiently to weaken central tolerance without producing overt immune deficiency at baseline. Such an effect could increase susceptibility to autoimmune responses after appropriate inflammatory or antigenic challenge.

No study has established in humans that mTEC-deficient thymic regions impair negative selection and thereby promote autoimmunity. Nevertheless, perivascular spaces (PVSs), which lack mTECs in thymic parenchyma, have been described in both mice and humans (59–61). In mice, PVSs serve as transit compartments where the most mature single-positive thymocytes accumulate immediately before vascular egress (19, 62). Perturbation of the S1P-S1PR1 axis (S1P lyase inhibition) causes mature thymocyte retention and PVS enlargement (20). Age-associated PVS expansion has been reported in human and mouse thymi (63, 64). These observations raise the possibility that rat mEFAs correspond to PVSs. Although direct evidence is lacking, PVS expansion might be either a cause or a consequence of thymic dysfunction: PVSs might expand as a result of impaired thymic egress, or expanded PVSs might compress functional mTEC-containing regions, thereby disrupting interactions between normal thymic stroma and SP thymocytes, ultimately compromising central tolerance. PVS expansion might therefore be associated with a partial failure of central tolerance in human disease, while such a defect could remain undetected during routine diagnostic evaluation.

Conversely, our data suggest that mEFAs do not simply represent dysfunctional medullary regions. First, we observed that TMC3/4 expressed LTβR-regulated TRAs (**Figure 3C**). Nitta et al. reported that mFbs express TRAs and that impaired maturation of these cells can lead to autoimmunity (6). In our previous study, we also found that, although reduced in number, DCs remained present in mEFAs at non-negligible levels (3). Thus, TRAs expressed by TMC subsets could potentially be transferred to and presented by DCs to SP thymocytes within mEFAs. Second, both rat mesenchymal subsets and SP thymocytes exhibited specialized transcriptional features that may preserve medullary functions despite the paucity of mTECs in mEFAs (**Figures 4A, B**). Predicted interactions between rat TMC3/4 subsets and SP thymocytes were more diverse than those detected between mouse medullary fibroblasts and SP thymocytes.

Several of these interactions may represent compensatory specialization associated with the mTEC-poor environment of mEFAs. For example, *Ccl21* (65, 66), *Icoslg* (67, 68), and *Il7* (69, 70), which contribute to stage-specific thymocyte positioning, thymocyte differentiation, negative selection, and survival in the medulla, were expressed by mTEC subsets and rat TMC3/4 but were not detected in the corresponding mouse mFb subsets (**Figure 4B**). This pattern suggests that TMC3/4 may partially complement functions normally provided by mTECs. In contrast, *Cxcl12*, laminins, and collagens were expressed by both mouse mFbs and rat TMC3/4. However, expression of their corresponding receptors, including *Cxcr4*, *Itgb1*, *Itga6*, *Cd44*, and *Sv2b*, was detected more prominently in rat SP subsets, generating predicted ligand-receptor interactions that were not detected in the corresponding mouse SP subsets. These interactions may promote localization or retention of mature SP thymocytes within mEFAs. Of particular interest, CXCL12-CXCR4 signaling is well established as a regulator of DP thymocyte positioning (65, 66). The expression of *Cxcr4* by rat SP subsets therefore raises the possibility that this positioning system is redeployed at a later developmental stage to facilitate SP thymocyte localization within mEFAs.

We included the mouse aaTEC2 subset in these analyses because of its proximity to the rat TMC4 cluster in the integrated UMAP (**Figure 3A**) and their shared expression of *Ccl21* and *Il7* (**Figure 4B**). The age-dependent expansion of mEFAs (**Figure 1A**) further suggested a possible relationship between TMC4 and aaTEC2, although mEFA formation begins too early to be considered a feature of senescence alone. We therefore hypothesized that TMC4 might represent a rat population related to mouse aaTEC2. However, neither expression of aaTEC2-associated genes nor analysis of epithelial**–**mesenchymal transition signatures revealed a clear transcriptional relationship between aaTEC2 and TMC4 (data not shown). Thus, the present transcriptomic data do not support a direct aaTEC2-TMC4 relationship, and determining whether these populations share a developmental origin will require lineage-tracing approaches in rats.

As stated in the Introduction, it has been reported that both mesenchymal (mFbs and pericytes) and EC subsets contribute to the S1P gradient that promotes mature-thymocyte egress (19, 20, 23–27). Consistent with this concept, we found that both TMC4 and vEC expressed the S1P transporter gene *Spns2*, whereas both capFb and capEC expressed the S1P phosphatase gene *Plpp3* (**Figures 4C**, **5D**). Because TMC4 and vEC were enriched in mEFAs, it is hypothesized that S1P-mediated thymic egress occurs in mEFAs, while S1P concentrations are kept low in capsular regions.

These findings raise the possibility that mEFAs contain exit-associated TPECs (**Figure 5**), whereas in mice, exit-associated blood vessels have been reported mainly at the corticomedullary junction (CMJ) (19, 28). In our material, mEFAs did not always coincide with the CMJ (**Figure 1E**); some appeared to be surrounded by mTECs. A similar pattern was reported in IL-4Rα-deficient mice, in which mEFA-like structures were also not restricted to the CMJ (47). In that study, increased labeling by intravenously injected anti-CD4 antibody in IL-4Rα-deficient thymi suggested accumulation of portal endothelial structures and mature thymocytes within mEFA-like regions, consistent with our findings. Together, these observations suggest that mEFAs containing TPECs can form not only near the CMJ but also deep within the medulla.

Given the broad genomic and cellular conservation among mammals, it is unlikely that mEFAs, their associated mesenchymal subsets, and their supporting gene programs are entirely unique to rats. Rather, mice and humans may also have the capacity to generate similar structures under altered signaling conditions, disease states, or senescence. Further investigation of mEFAs and their localized stromal niches may therefore improve our understanding of medullary thymic organization, thymic egress, and the mechanisms linking stromal architecture to immune tolerance and autoimmunity.

## 5 Study limitations

This study has several limitations. First, the Stereo-seq dataset and the rat thymocyte and stromal scRNA-seq datasets were each generated from a single, distinct Lewis rat. Therefore, interindividual variability in the transcriptomic and spatial features identified here could not be assessed; only histological variation in mEFA distribution across ages and rat strains was evaluated. Moreover, all rat omics datasets were derived from Lewis rats, whereas most cells in the mouse reference datasets were derived from C57BL/6 mice or C57BL/6-based transgenic lines. Consequently, some cross-species differences in gene expression and predicted cell–cell interactions may depend on genetic background, and their generalizability to other rat and mouse strains remains to be established. Nevertheless, several major observations were supported by concordant analyses across independent data modalities, including spatial localization, scRNA-seq-derived transcriptional states, and immunohistochemistry. Second, the proposed functional properties of TMC3/4 and endothelial subsets, including their predicted interactions with thymocytes and possible involvement in S1P-mediated thymic egress, were inferred mainly from gene-expression profiles and computational analyses. Direct functional perturbation will be required to establish these mechanisms. These limitations should be considered when interpreting the mechanistic implications of the present findings.

## Data Availability

Data supporting this study have been deposited in the Gene Expression Omnibus under accession number GSE333693, including rat thymic stromal and thymocyte scRNA-seq datasets and a spatial transcriptomics dataset from a rat thymic frozen section. The deposited files include scRNA-seq expression matrices, Seurat object metadata, the.loom file used for RNA velocity analysis, h5ad files for spatial transcriptomics, and the.stereo file for StereoMap. The mass spectrometry proteomics data and results have been deposited in the jPOST repository (71, 72) with the dataset identifier JPST004670. R and Python scripts are available on GitHub at https://github.com/yasuhisawanobori/rat_mEFA_spatial_analysis. This study also used published scRNA-seq datasets for mouse thymocytes (GSE186078) and mouse thymic stroma (https://zenodo.org/records/12516405).

## Supplementary material

Supplementary Figure 1 Clustering and annotation of the rat thymocyte scRNA-seq dataset. **(A)** A UMAP plot and a dot plot of representative markers of the rat thymocyte scRNA-seq dataset. An initial clustering analysis was performed. **(B)** A UMAP plot of a mouse thymocyte scRNA-seq dataset. Expression matrices from multiple individuals were integrated with the JointPCAIntegration algorithm. Clustering was performed following the original study with some modifications. **(C)** Distributions of cells from CD4-fated and CD8-fated transgenic mice, and expression of representative marker genes for CD4^+^ and CD8^+^ SP subsets. **(D)** Expression of marker genes in the mouse thymocyte scRNA-seq dataset. **(E)** UMAP plots of the cross-species integrated scRNA-seq datasets of thymocytes. Datasets were integrated with the JointPCAIntegration algorithm, and mouse clustering was projected onto rat cells. DN = double-negative thymocyte, DP-P = double-positive proliferating thymocyte, DP-sig = DP thymocytes that had received the positive-selection signal. SP = single-positive thymocyte. intCD4 = intermediate CD4^+^ SP thymocyte. AgoSel = agonist-selected subset. gdT = gamma-delta T cell.

Supplementary Figure 2 Clustering and annotation of the rat scRNA-seq dataset of thymic stroma. **(A)** A UMAP plot and a dot plot of representative markers in the rat scRNA-seq dataset of thymic stroma. An initial clustering analysis was performed. **(B)** UMAP plots of the cross-species integrated scRNA-seq datasets of thymic stroma. Datasets were integrated with the CCAIntegration algorithm, and mouse clustering was projected onto rat cells. **(C)** Pearson correlation coefficients represent similarities between mouse and rat thymic stromal subsets. Endo = endothelial subset. See the original study for abbreviations in the mouse stromal cell dataset (34).

Supplementary Figure 3 RCTD-based prediction of cell subsets. **(A)** A UMAP plot of the integrated scRNA-seq datasets of rat thymic stromal cells and thymocytes. The datasets were integrated with the JointPCAIntegration algorithm. **(B)** The number of predicted segments in the CellBin and Bin20 ST datasets. The top three predictions are displayed. Note that y-axes are logarithmic. **(C)** TEC-, TMC-, and EC-associated genes detected in RCTD-predicted thymocytes. Segments predicted as thymocytes in mECAs and mEFAs were compared using the FindMarkers() function. Representative enriched genes and their expression in the reference scRNA-seq dataset are displayed. **(D)** Pictorial representations of ssDNA staining-based segmentation and equal segmentation. Arrows indicate fibroblast and TEC protrusions whose transcripts may be assigned to neighboring segments.

Supplementary Figure 4 Distribution of TEC subsets in the ST dataset and investigations into the iNKT–type 2 cytokine–TEC axis. **(A)** Distribution of RCTD-predicted segments corresponding to TEC subsets in the ST dataset. Numbers in the heatmap indicate the percentage in each region, whereas colors correspond to z-scores across regions. **(B)** Expression of type 2 cytokine genes and ratios of NKT cells in mouse and rat thymocyte subsets. **(C)** Expression of IL-4 receptor and signaling components in TEC subsets. early Pr = early TEC progenitor. mTEC-prol = proliferating mTEC.

Supplementary Figure 5 Identification of candidate ED21 target proteins. **(A)** ED21 staining pattern reported in our previous study, and MHCII- and *Aire*-related genes in TEC clusters. **(B)** Schematic overview of the workflow used to narrow down candidate ED21 target proteins. **(C)** Expression of LC-MS/MS-screened candidate genes in the rat scRNA-seq reference dataset. **(D)** Spatial expression of candidate genes that showed TEC-associated expression in panel C, visualized with StereoMap. Arrows indicate *Krt14* expression in capsular regions. **(E)** Immunohistochemical staining of capsular regions in the thymus of a 5-week-old male rat. Arrows indicate ED21 staining along an interlobular capsule. **(F)** Expression of *Krt14* orthologs and paralogs in the mouse and rat scRNA-seq datasets. **(G)** Protein sequence alignment of mouse and rat keratins 14 and 17.
